# Sleeping for two: study protocol for a randomized controlled trial of cognitive behavioral therapy for insomnia in pregnant women

**DOI:** 10.1186/s13063-021-05498-w

**Published:** 2021-08-12

**Authors:** Anna L. MacKinnon, Joshua W. Madsen, Ashley Dhillon, Elizabeth Keys, Gerald F. Giesbrecht, Tyler Williamson, Amy Metcalfe, Tavis Campbell, Kelly J. Mrklas, Lianne Tomfohr-Madsen

**Affiliations:** 1 University of Calgary, Calgary, Canada; 2 University of British Columbia, Okanagan Campus, Canada; 3 Alberta Health Services, Calgary, Canada

**Keywords:** CBT, Insomnia, Pregnancy, Sleep, Therapy, RCT

## Abstract

**Background:**

Insomnia and sleep disturbances are common in pregnancy and have potentially significant consequences for both maternal and infant health. There is limited research examining the effectiveness of cognitive behavioral therapy for insomnia (CBT-I) during pregnancy. With increased distress and limited access to services during the COVID-19 pandemic, there is also an unprecedented need for telehealth delivery of treatment programs for pregnant women. The aims of this trial are to evaluate the impact of the Sleeping for Two adaptation of CBT-I in pregnancy (in-person or telehealth) versus treatment as usual (TAU) in reducing symptoms of insomnia (primary outcome), as well as increasing gestational length and reducing symptoms of depression (secondary outcomes).

**Methods:**

A two-arm, single-blinded, parallel group randomized controlled trial (RCT) design with repeated measures will be used to evaluate the impact of CBT-I compared to TAU among a sample of 62 pregnant women, enrolled between 12 and 28 weeks of gestation, who self-identify as experiencing insomnia. Five weekly individual sessions of CBT-I will be delivered in person or via telehealth depending on physical distancing guidelines. Assessment of insomnia diagnosis by structured interview, self-reported insomnia symptom severity and sleep problems, and sleep quantity and quality as measured by a daily diary and actigraphy will occur at 12–28 weeks of pregnancy (T1), 1 week post-treatment (T2), and 6 months postpartum (T3).

**Discussion:**

CBT-I delivered in pregnancy has the potential to reduce symptoms of insomnia and depression and could lead to reduced risk of preterm birth, all of which can minimize risk of negative maternal and child health and developmental consequences in the short (e.g., infant death) and long terms (e.g., developmental delays). This RCT builds on a successful open pilot trial conducted by our team and will provide further evaluation of a novel evidence-based treatment for pregnancy-related insomnia, which can be widely disseminated and used to treat individuals that are most in need of intervention. Findings will enhance understanding of pregnancy-related sleep problems, as well as means by which to improve the health and sleep of mothers and their children.

**Trial registration:**

ClinicalTrials.gov NCT03918057. Registered on 17 April 2019.

## Administrative information

Note: the numbers in curly brackets in this protocol refer to SPIRIT checklist item numbers. The order of the items has been modified to group similar items (see http://www.equator-network.org/reporting-guidelines/spirit-2013-statement-defining-standard-protocol-items-for-clinical-trials/).

**Table:** 

Title {1}	Sleeping for Two: study protocol for a randomized controlled trial of cognitive behavioural therapy for insomnia in pregnant women
Trial registration {2a and 2b}.	The trial was registered with ClinicalTrials.gov (NCT03918057) on April 17, 2019.
Protocol version {3}	April 15, 2020 (Version 4)
Funding {4}	This work was supported by an Early Career Investigator operating grant in Maternal, Reproductive, Child & Youth Health from the Canadian Institutes of Health Research (LTM).
Author details {5a}	Anna L. MacKinnon, University of Calgary Joshua W. Madsen, University of Calgary Ashley Dhillon, University of Calgary Elizabeth Keys, University of British Columbia, Okanagan Campus Gerald F. Giesbrecht, University of Calgary Tyler Williamson, University of Calgary Amy Metcalfe, University of Calgary Tavis Campbell, University of Calgary Kelly J. Mrklas, Alberta Health Services Lianne Tomfohr-Madsen, University of Calgary
Name and contact information for the trial sponsor {5b}	Lianne Tomfohr-Madsen Department of Psychology, University of Calgary 2500 University Drive NW, Calgary, Alberta, T2N 1N4
Role of sponsor {5c}	This funding source had no role in the design of this study and will not have any role during its execution, analyses, interpretation of the data, or decision to submit results.

## Introduction

### Background and rationale {6a}

Insomnia and poor sleep quality—defined as difficulty initiating or maintaining sleep, restriction in hours of sleep, or feeling that sleep is unrefreshing—is common in pregnancy [1, 2]. Meta-analyses suggest that the prevalence of poor sleep quality and clinically significant insomnia during pregnancy ranges from 20 to 60%, depending on trimester and method of assessment [3, 4]. Since being declared a global pandemic on March 11, 2020 [5], the outbreak of coronavirus disease 2019 (COVID-19) and the related public health measures put in place to limit its spread (i.e., physical and social distancing) have been associated with significantly increased distress and mental health concerns, particularly among pregnant women [6, 7]. Not surprisingly, sleep has also been negatively impacted; emerging evidence suggests an increase in sleep disturbances among adults during the pandemic, including poor sleep quality and symptoms of insomnia [8, 9], particularly among females [10]. Pregnant women are reporting an average of 6.97 h of sleep per night during the pandemic (Lebel et al., unpublished data), which falls slightly below the National Sleep Foundation’s recommendation of ≥7 h among adults [11], and below the 8.2 h, pregnant women have reported they need to feel rested [12].

Unfortunately, insomnia and poor sleep quality during pregnancy have serious consequences for both maternal and child health, including associations with shorter gestational age (i.e., preterm birth) [13–18]. In turn, these poor birth outcomes confer long-term infant and child health risks [19–21]. Symptoms of insomnia in pregnant and non-pregnant populations are a robust predictor of increased risk of developing symptoms of depression [22–28]. In the first analysis of sleep trajectories during pregnancy, our research team showed that trajectories characterized by consistently poor sleep quality in the perinatal period or in the third trimester of pregnancy were associated with a 10- to 20-fold increased risk of postpartum depression (PPD), even after controlling for robust predictors such as depression in pregnancy and socioeconomic status [29]. Some research has also shown sleep disturbances predate increases in anxiety during pregnancy [27, 30]. Without intervention, symptoms of depression and anxiety are relatively stable from pregnancy to the postpartum period [31], and associated with serious long-term negative consequences for child development across physical, social, emotional and cognitive domains [32–35]. Interventions targeting antenatal sleep may prevent the direct consequences of poor sleep on maternal-child health and also prevent or minimize the myriad negative child developmental consequences associated with PPD and postpartum anxiety.

Poor sleep quality and maladaptive maternal cognitions about sleep have been shown to predict poor infant sleep [36, 37]. Poor infant sleep-wake organization contributes to child developmental outcomes, such that unconsolidated and short sleep in infancy and childhood can significantly interfere with attention regulation, concentration, and cognitive, social and behavioural development [38–49]. The largest changes in sleep maturation are observed in the first six months of life, after which point changes continue to occur but at a more moderate pace [50, 51]. At six months of age, sleep has consolidated to the point where many infants are considered to be “sleeping through the night” [50–53]. However, 20-30% of typically developing infants continue to experience multiple extended nighttime awakenings [54, 55]. Optimizing sleep in an at-risk group of mothers suffering from antenatal insomnia is an important and understudied means of potentially improving both maternal health and child development.

The prevalence and consequences of untreated antenatal insomnia for maternal mental health and infant development highlight the need for evidence-based treatment for insomnia during pregnancy. Additionally, patient-oriented approaches to research priority-setting (from conception to age two) show that parents, clinicians, and policymakers rate optimizing sleep quality as a top research priority [56]. Use of pharmacotherapy in pregnancy may improve sleep quality [57]; however, best practice guidelines advise against the use of the majority of sedative sleep medications in pregnancy due to potential teratogenicity [58]. Additionally, pregnant women are reluctant to take prescription medications due to their perception of risk for the developing fetus [59, 60]. In two studies of treatment preferences for insomnia during pregnancy, our team found that when presented with descriptions of pharmacotherapy versus cognitive behavioural therapy for insomnia (CBT-I), pregnant women and their partners rated CBT-I as the more credible and preferable treatment [61, 62]. Matching patients to their preferred treatment modality increases adherence and treatment response [63].

CBT-I is an evidence-based psychotherapeutic intervention that combines cognitive and behavioural principles. Psychoeducation about thoughts that contribute to the maintenance of sleep problems is provided, and instruction in behavioural techniques is offered to help decrease sleep onset latency and promote sleep maintenance. CBT-I is an effective treatment for insomnia, with short-term efficacy equivalent to medication and long-term results showing that it outperforms medication in improving sleep, while also conferring a secondary benefit of reducing symptoms of depression [64–67]. In fact, CBT-I, not sedative medication, is the current gold standard for treatment of insomnia (American Academy of Sleep Medicine) [68]. However, pregnant women have historically been excluded from intervention research—and insomnia treatment is no exception—which prompted calls for further development of interventions to treat insomnia in pregnancy and the postpartum period [69].

There have been four studies to date [70–73] examining the effectiveness of in-person or digital CBT-I specifically in pregnancy, all showing positive results in favour of active intervention. Following the ORBIT model [74, 75], our research team conducted a Phase II open pilot trial of CBT-I for pregnant women with insomnia, demonstrating that it was effective in reducing both subjective symptoms of insomnia and improving objective indices of sleep quality [70]. Another research group conducted a multi-site randomized controlled trial (RCT) of CBT-I with an ethnically diverse sample of treatment naive women [71]. Results indicated reductions in insomnia severity, self-reported total wake time, and symptoms of depression. Two RCTs have examined the efficacy of digital CBT-I in pregnancy delivered online, with results indicating that a digital CBT-I program Sleepio (Big Health Inc.) was associated with reductions in insomnia severity and improvements in sleep duration and quality [72, 73].

With limited access to in-person services and increased fear of threat to the health of their baby during the COVID-19 pandemic [6], there is an unprecedented need for telehealth delivery of programs for pregnant women. Telehealth refers to the provision of health care services using technological modalities instead of, or in addition to, in-person methods, and include behavioural and/or mental health care (e.g., therapy using the phone, diagnostic interviews via videoconferencing, apps to track mood states, and email consultations) [76]. Telehealth adaptations of CBT-I also address barriers to accessing treatment reported by pregnant women, such as transportation, childcare and/or work hours [77–80]. The COVID-19 pandemic has served as a catalyst for the implementation of telehealth mental health care services [81]. Indeed, our team launched a Phase III randomized controlled trial of CBT-I for pregnant women comprising five individual in-person therapy sessions, however, when COVID-19 was declared a pandemic and physical distancing measures were enacted, we amended the protocol to deliver the intervention by telehealth (i.e., videoconferencing). This presents the unique opportunity to be the first study to investigate the efficacy of CBT-I delivered via telehealth during pregnancy.

## Objectives {7}

The primary aim of this study is to conduct a phase III RCT to evaluate the impact of CBT-I for treatment of insomnia experienced in pregnancy compared to a treatment as usual (TAU) control group. We hypothesize that participants who receive the CBT-I intervention, delivered in person or via telehealth, will report fewer insomnia symptoms and have improved objectively assessed sleep measured at 1 week post-treatment (T2), and that treatment gains will be maintained at 6 months postpartum (T3).

The secondary aims of the trial are to determine whether CBT-I versus TAU is associated with: (1) reduced symptoms of depression at T2 and T3; (2) and a lower risk of preterm birth as assessed by gestational length. We hypothesize that participants who receive CBT-I (versus TAU) will report fewer depressive symptoms at 1 week post-treatment (T2) and that symptom reduction will be maintained at 6 months postpartum (T3). We also hypothesize that CBT-I versus TAU will be associated with a lower risk of preterm birth (confirmed by public health records after delivery, between T2 and T3).

Exploratory research questions will investigate whether CBT-I delivered in pregnancy is associated with: (1) fewer symptoms of maternal anxiety at T2 and T3; and (2) better infant sleep at 6 months postpartum (T3). We hypothesize that mothers who receive CBT-I compared to the TAU group will report lower anxiety at 1 week post-treatment (T2) and that treatment gains will be maintained at 6 months postpartum (T3). We also hypothesize that the infants of mothers who receive CBT-I will demonstrate better sleep at 6 months. Additionally, we expect CBT-I in pregnancy delivered via telehealth will be associated with the same treatment response outcomes as in-person therapy, given both use the same protocol for model fidelity (i.e., manualized, supervision, monitoring).

## Trial design {8}

A two-arm, single-blinded, parallel group randomized controlled trial (RCT) design with repeated measures will be used to evaluate the impact of CBT-I delivered in person or via telehealth for treatment of insomnia experienced in pregnancy compared to the TAU control group. Assessments will occur at baseline, between 12 and 28 weeks of pregnancy (T1), 1 week post-treatment (T2), and 6 months postpartum (T3).

This trial adheres to the Standard Protocol Items: Recommendations for Intervention Trials (SPIRIT) guidelines [82] and was registered with ClinicalTrials.gov (NCT03918057). All procedures were performed in accordance with the University of Calgary Conjoint Health Research Ethics Board (REB19-0465) and the 1964 Helsinki declaration and its later amendments. All participants provided informed consent prior to enrollment in the study.

## Methods: participants, interventions, and outcomes

### Study setting {9}

Participants will be recruited from Alberta, Canada. Data will be collected in-person at the University of Calgary or online through Qualtrics® survey software (SAP America Inc.), which is hosted on the university’s secure server. Therapy was being conducted in-person at the University of Calgary Psychology Clinic prior to COVID-19 being declared a pandemic and then via telehealth after physical distancing measures were implemented, using the clinic’s securely hosted Jane App (Jane Software Inc.) online platform for virtual health appointments.

### Eligibility criteria {10}

#### Inclusion and exclusion criteria

English speaking women from Alberta, over the age of 18, who are 12–28 weeks of gestation, and who report experiencing insomnia in pregnancy will be eligible. Exclusion criteria include the following: (1) experiencing symptoms of sleep disorders other than insomnia (i.e., restless legs syndrome, sleep-disordered breathing); (2) having a lifetime diagnosis of bipolar or psychotic disorder; (3) currently taking prescribed medications for sleep problems or taking medications known to impact sleep; (4) smoking, drinking alcohol, or drug abuse during pregnancy; (5) pregnant with multiples; and (6) diagnosis of chronic pain.

#### Screening and enrollment

After informed consent is obtained, participants answer a series of questions during a phone screen; those above the cut-off score of 10 on the Insomnia Severity Index (ISI), which yields 86.1% sensitivity and 87.7% specificity for detecting insomnia in a community sample [83], will be invited to complete a subsequent baseline assessment, in the lab or via online videoconference, to determine further eligibility for the study. Insomnia diagnosis will be confirmed, and screening for restless legs syndrome (RLS) and sleep-disordered breathing (SDB) will be conducted using the Structured Clinical Interview for DSM-5 Sleep Disorders (SCISD) [84]. Women whose scores are suggestive of a sleep disorder other than insomnia will be referred to their primary care provider for further assessment. Participants will also be administered the Diagnostic Interview for Anxiety, Mood, and OCD and Related Neuropsychiatric Disorders (DIAMOND) [85], to assess if any other exclusionary criteria are present. Diagnoses will be confirmed in consensus conferences with a licensed, doctoral level clinical psychologist.

### Who will take informed consent? {26a}

Prospective participants who agree to be involved in the study will meet with a member of the research team, who will explain the study procedures in full detail and obtain written consent.

### Additional consent provisions for collection and use of participant data and biological specimens {26b}

Not applicable.

## Interventions

### Explanation for the choice of comparators {6b}

The control arm of the study is designed to account for the potential effects of time and regular care on the change in insomnia symptoms [74]. A control group will be used to test whether CBT-I will result in improvement in study outcomes (sleep, mood) compared to treatment as usual (TAU). At the end of their participation (completion of follow-up visit), TAU/control participants will be offered the CBT-I intervention, which has been successfully delivered in the postpartum [86].

### Intervention description {11a}

The Sleeping for Two adaptation for CBT-I in pregnancy consists of five weekly 60-minute individual therapy sessions of CBT-I. Participants are asked to use a daily sleep diary for self-monitoring and receive a manual containing readings summarizing the material being taught in-session. The manual was adapted from a treatment protocol previously validated for oncology patients with comorbid insomnia [87]. In adapting the CBT-I protocol for use with pregnant women, we followed steps outlined in the ORBIT Model [74, 75] for developing behavioural therapies to treat or prevent chronic diseases and enhance health-promoting behaviours (e.g., sleep): Phase I (i.e., defined the question), Phase IIa (i.e., created a fixed treatment package; see Appendix A for session structure), Phase IIb (i.e., pilot tested the intervention), and Phase III (i.e., a randomized controlled trial; RCT). This manuscript represents Phase III.

During this five-session intervention, patients are familiarized with the self-management approach for insomnia (e.g., active vs. passive coping strategies). First, patients are oriented to CBT-I (e.g., use of daily sleep diaries) and set individualized treatment goals (Session 1). The remaining sessions consist of weekly review of sleep diaries, psychoeducation about sleep and sleep consolidation (Session 2), stimulus control (Session 3), cognitive strategies to examine attitudes and beliefs about sleep (Session 4), and problem-solving to cope with sleep disturbances and relapse prevention (Session 5). In the final session, participants are also provided information on infant sleep and the importance of mutual regulation, which was adapted from evidenced-based behavioural-education programs [88] and consultation with infant sleep experts. A licensed clinical

psychologist with extensive training in CBT-I will supervise the administration of the intervention (LTM).

The control group will receive regular obstetric care provided according to the needs and current regime of the patient. The amount of contact with health care professionals outside of the study will be recorded, coded, and reported, as well as any treatment for insomnia (e.g., medication use, searching the internet for techniques to improve sleep) that the patient engages in. After study completion, TAU participants will be offered the CBT-I intervention, which has been successfully delivered in the postpartum [86].

### Criteria for discontinuing or modifying allocated interventions {11b}

Participants who do not appear for the first or second session are not able to continue receiving treatment (but can remain in the study). In accordance with the College of Alberta Psychologists Practice Guidelines for Telepsychology Services, therapists may decide to terminate telehealth services if they deem it inappropriate for the client to continue therapy through videoconferencing sessions (e.g., the participant cannot ensure privacy and confidentiality is maintained through availability of a secure space and stable Internet connection, or environmental factors interfere with their engagement). In this case, the investigators will provide in-person care or a referral to another provider or clinic, if necessary.

### Strategies to improve adherence to interventions {11c}

Scheduled study-related reminders will be sent to participants through the Jane App service. Sleep diaries and homework for the past week will be reviewed at the beginning of each session. Therapists will also be instructed to use several strategies to promote treatment adherence, including 5-step problem-solving, identifying barriers and resistance, reframing, cognitive restructuring, and reminding patients of the goals they set at the beginning of treatment.

### Relevant concomitant care permitted or prohibited during the trial {11d}

Potential participants who report taking prescribed medications for sleep problems will not be invited to participate in the study due to potential side effects of medication during pregnancy. Because this intervention is designed to be an adjunct to usual care in pregnancy, participants will not be excluded if they are receiving common medical and physical therapies that are not known to interfere with sleep. Participants will be permitted to receive concomitant care for unrelated mental health problems (i.e., not insomnia).

### Provisions for post-trial care {30}

At the end of their participation (completion of follow-up visit), TAU/control participants will be offered CBT-I treatment. If further treatment is deemed necessary, a referral to another provider or clinic will be made, and a list of community services will be provided.

### Outcomes {12}

The primary outcome is mean change in insomnia severity across all timepoints. The mean change in sleep quality (measured by self-report questionnaires) and sleep parameters such as onset latency, wake after sleep onset, number of awakenings, total sleep time, and sleep efficiency (measured by actigraphy and daily sleep diaries), as well as insomnia diagnosis remission, will also be evaluated.

Secondary outcomes include mean change in symptoms of depression (measured by self-report questionnaire) across all timepoints, as well as the rate of preterm birth (assessed from public health records at T3).

Exploratory outcomes include mean change in symptoms of anxiety (measured by self-report questionnaires) across all timepoints, as well as mean infant sleep problems (measured by parent-report questionnaire at T3).

### Participant timeline {13}

**Fig. 1:**
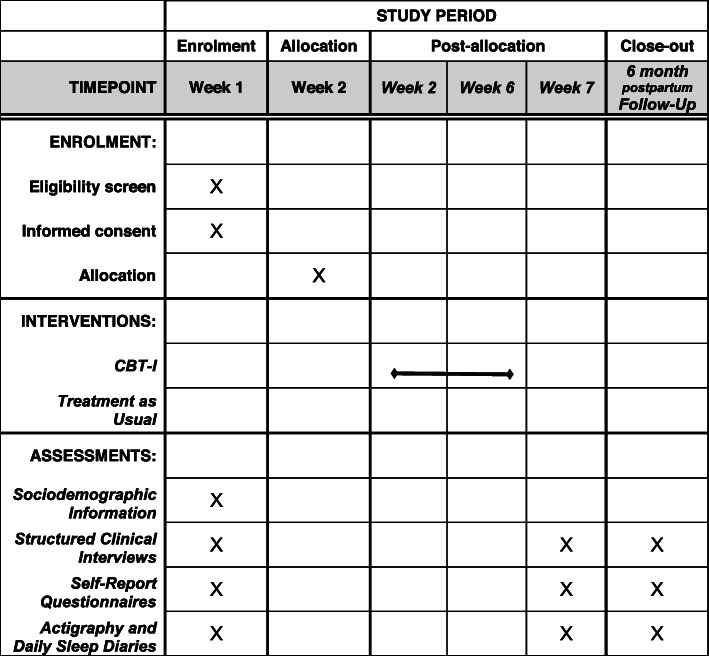
SPIRIT schedule of enrolment, intervention, and assessments

The overall participant timeline is illustrated in Fig. 1.

Week 1 (T1): Eligible participants will meet with a member of the research team at the laboratory or via an online assessment hosted using the Jane App, who will explain the study protocols and obtain consent (either written or via Qualtrics). Participants will be asked to complete the series of structured clinical interviews, self-report questionnaires via Qualtrics, and then are provided with an actigraphy device and a link to an online daily sleep diary to complete for a period of 7 consecutive days.

Week 2: Participants will be randomly allocated to CBT-I or TAU.

Weeks 2 to 6: If randomized to the treatment group, participants will be invited to attend 5 sessions of CBT-I in person or via telehealth. Participants in the TAU group will receive regular obstetric care provided according to the needs and current regime of the patient.

Week 7 (T2): After 7 weeks, all the participants will be invited to complete the same set of questionnaires from the baseline assessment. Additionally, participants will be sent an Actiwatch and a link to an online daily sleep diary to complete for a period of 7 days.

Six months postpartum (T3): At 6 months postpartum, all participants will be invited to a web-link with the same set of questionnaires from the previous points of assessment, as well as one additional questionnaire assessing infant sleep quality. Participants will also be sent an actiwatch and a link to an online daily sleep diary to complete for a period of 7 days.

### Sample size {14}

We used pre-post data available from our Phase IIb pilot trial, comparing in-person CBT-I to waitlist control (*η*^2^ = .41, *f* = 0.84), to conduct a power analysis. The effect size was large, and the power analysis indicated that we would need 18 participants to provide power over 90% to detect a clinically significant change of 8 points on the ISI [83]. Although longitudinal analyses of CBT-I show that gains are maintained up to two years posttreatment, we wanted to account for the possibility that the effects of treatment may decrease by 6 months postpartum. We therefore powered the RCT to detect a medium effect size (*﻿f* = 0.25), resulting in a total sample size of 44 participants (22 per group) over three assessment time points. Although retention in our studies has been excellent, we accounted for a potential attrition rate of 20% based on previous research in this area with longer follow-up periods [89], resulting in an initial total of 54 participants. When the protocol was amended due to the COVID-19 pandemic, 2 participants had to drop out of the intervention due to pandemic related schedule adjustments and we were unable to send actiwatches to 3 participants due to the temporary university closure. Therefore, the recruitment of 5 additional participants was needed to make up for the incomplete data and/or participation. To ensure there was a 1:1 ratio of participants per group, a final total of 62 participants will be recruited.

### Recruitment {15}

Participants will be recruited from Alberta, Canada, through ultrasound clinics and a low risk primary care maternity clinic, where they will be approached by a research assistant or can respond to the posted advertisements. Potential participants will also be able to self-refer through advertisements posted online (e.g., lab website and social media accounts including Facebook, Instagram, and Twitter). Those interested in participating will contact the investigators’ lab and receive additional information about the study protocols.

## Assignment of interventions: allocation

### Sequence generation {16a}

Participants will be randomly allocated in a 1:1 ratio to CBT-I or TAU. Randomization will be determined using an online research randomization tool (GraphPad Software), which is not predictable and eliminates potential experimenter bias [90]. Sixty-two participants will be enrolled in the RCT, with 31 randomized into each study group. 

### Concealment mechanism {16b}

Participant assignments will be placed into opaque envelopes that contain the results of the randomization.

### Implementation {16c}

A staff member who is not associated with the study will conduct the randomization and sequence generation. After the baseline assessment is completed, a research assistant will open the envelope and inform participants of their assignment.

## Assignment of interventions: blinding

### Who will be blinded {17a}

Participants and therapists will not be blinded to condition. The research assistants conducting the clinical interviews, administering psychological questionnaires, and coordinating completion of the actigraphy and daily diary assessments will be blinded to participants’ group assignment, as will be the psychologists supervising the follow-up diagnostic interviews.

### Procedure for unblinding if needed {17b}

Not applicable.

## Data collection and management

### Plans for assessment and collection of outcomes {18a}

#### Primary outcome

Self-reported measures of sleep are considered the gold standard for the primary outcome in clinical trials, as they are often more sensitive to treatment effects [91]. Insomnia symptoms will be assessed using the Insomnia Severity Index (ISI). The is a 7-item questionnaire designed to identify cases of insomnia and evaluate treatment outcomes [83]. The ISI assesses severity of sleep onset, sleep maintenance and early wakening problems, sleep dissatisfaction and perceived distress caused by sleep problems [92]. Higher scores on the ISI are indicative of more symptoms of insomnia [93]. The ISI has been reliably used to assess insomnia and response to CBT-I treatment during pregnancy [70, 71, 86].

#### Insomnia diagnosis

Insomnia diagnosis will be confirmed using the Structured Clinical Interview for DSM-5 Sleep Disorders (SCISD) [84], which produces reliable and valid insomnia diagnoses, and provides information about developmental course and impact [94].

#### Objective sleep measure

Objective sleep quantity and quality will be assessed using actigraphy (Actiwatch II, Phillips, USA). Actigraphy monitoring provides objective information on circadian rhythm amplitude, acrophase, and mesor as well as indices of sleep efficiency, sleep latency, total sleep time, and number and frequency of awakenings [95]. Actigraphy has been used in research involving pregnant women, mothers, and infants, and is considered a valid measure of sleep quality and quantity [96, 97] as it yields estimates that are highly correlated with polysomnography [98]. Consistent with actigraphy recommendations, participants will be asked to continuously wear the actigraph for seven days after each in-person assessment point [99, 100].

#### Patient-reported sleep measures

Daily sleep diary logs will be used to measure total sleep time, time in bed, sleep efficiency, sleep onset latency, and number of awakenings [101]. Sleep diaries will be used to verify actigraphy data, and changes will be made to sleep intervals only when a manual calculation error was observed, for example, if a rest interval was identified when a participant indicated that she had not been wearing the watch. The Pittsburgh Sleep Quality Index (PSQI) is a 19-item instrument assessing sleep quality during the previous month [2]. There are seven components of the PSQI and these are subjective sleep quality, sleep latency, sleep duration, habitual sleep efficiency, sleep disturbances, use of sleeping medications and daytime dysfunction. A score above 7 has been used in other studies during pregnancy to indicate poor subjective sleep quality [15]. The PSQI has been validated against polysomnography and used reliably among pregnant women [102–104].

#### Secondary outcomes

*Symptoms of depression* will be assessed with the Edinburgh Postpartum Depression Scale (EPDS) [105]. The EPDS consists of 10 items, and is a reliable and valid tool for identifying symptoms of depression in pregnancy and the postpartum [106]. We will also collect information about psychiatric diagnoses from the SCID-5 [107].

*Infant birth outcomes*, including information about preterm birth (e.g., gestational length), delivery type, and birth weight, will be gathered through linkage to participants’ heath records for the Alberta Perinatal Health Program.

#### Exploratory outcomes

##### Symptoms of anxiety

The Pregnancy Specific Anxiety (PSA) measure assesses the degree to which pregnant women have felt anxious, concerned, afraid, and panicky in the preceding week [108, 109]. The PSA is a reliable and valid indicator of anxiety about pregnancy-related issues, with strong face validity and predictive validity, and will be used to assess anxiety experienced during pregnancy [108, 109]. The Strait Trait Anxiety Inventory (STAI-6) will be used to measure both state anxiety (around a specific situation) and trait anxiety (as a personality trait), with acceptable reliability and validity [110].

##### Infant sleep

Subjective ratings of infant sleep will be obtained by maternal report using the Brief Infant Sleep Questionnaire (BISQ). The BISQ asks parents about their infant’s regular sleep patterns so an average sleep pattern for the infant can be determined [111]. This questionnaire has shown high test-retest correlations (*r *> .82) and been found to be highly correlated with measures such as sleep diaries and actigraphy [111].

#### Descriptive measures

Data will be collected regarding demographic and psychosocial variables known to impact sleep. Demographic information collected will include age, family socioeconomic status, and number of children at home. Health practices assessed include smoking, alcohol consumption, and level of habitual exercise. Assessment of previous mental health problems, sleep disorders, and psychotropic medication use will be assessed via interview.

### Plans to promote participant retention and complete follow-up {18b}

Research assistants will contact participants to book assessments. Scheduled study-related reminders for assessments and therapy sessions will be sent to participants through the Jane app service.

### Data management {19}

Data will be stored on a secure server in accordance with the University of Calgary’s Data Retention Policy. Only the primary investigator and study coordinators will be given access to the study folders where the database is saved.

### Confidentiality {27}

Patient confidentiality will be protected through all phases of assessment, treatment, and data analysis in accordance with University of Calgary ethics guidelines. All study personnel will sign a confidentiality/non-disclosure agreement form prior to their involvement in the study.

Participants will be forewarned that absolute confidentiality cannot be provided in a lab setting or online; others present may recognize the fact of their research participation, and information transmitted via the internet may not be completely secure. However, individual data provided by participants will be de-identified and anonymized to protect their privacy and confidentiality. Any documents with identifiable information (e.g., consent forms, case notes, contact information) will be password protected and/or stored in a locked, secure area at the University of Calgary separately from anonymized data. We have also implemented telehealth policies in accordance with the College of Alberta Psychologists Practice Guidelines governing the delivery of Telepsychology Services [112]. Participants will become familiar with the limits to confidentiality during the informed consent process before participating in this study.

For purposes of reliability and supervision, in-person therapy sessions may be audio and/or recorded, with participants’ permission. The contents of the taped sessions are confidential and they will be stored in a secure location and will not be used for any other purpose. The tapes will be erased after they have served their purpose. The recordings will be viewed only by the licensed clinical psychologist who will supervise the administration of the intervention and the team of graduate level practicum students who will deliver in-person therapy.

### Plans for collection, laboratory evaluation, and storage of biological specimens for genetic or molecular analysis in this trial/future use {33}

Not applicable.

## Statistical methods

### Statistical methods for primary and secondary outcomes {20a}

Categorical variables will be analyzed using chi-square tests. Ordinal variables will be analyzed using nonparametric tests in addition to using Student’s *t* tests. Independent-samples*t* test, *χ*^2^ test, and Fisher’s exact test will be used to compare the groups on demographic characteristics known to influence sleep, such as age, parity, body mass index, and socioeconomic status; covariates found to differ between groups will be controlled for in subsequent analyses. Primary analyses of insomnia severity will be conducted using generalized estimated equations (GEE) and an intent-to-treat (ITT) approach. Other sleep measures including insomnia diagnosis, self-reported sleep quality, and actigraphy parameters will also be assessed using GEE. Secondary response variables including diagnosis and symptoms of depression, as well as preterm birth, will be examined. Changes in binary outcomes will be tested using GEE with a binary outcome specified.

### Interim analyses {21b}

No interim analyses are planned and no interim data will be shared.

### Methods for additional analyses (e.g., subgroup analyses) {20b}

For exploratory outcome variables, symptoms of anxiety will be assessed by GEE, and differences in infant sleep measures, measured only at postpartum, will be assessed using ANOVA.

### Methods in analysis to handle protocol non-adherence and any statistical methods to handle missing data {20c}

Analyses will be conducted on an intent-to-treat (ITT) basis, including data for each participant who was randomized, regardless of their adherence to the intervention or whether they withdraw from the study. Analyses including only patients completing the treatment will also be conducted.

### Plans to give access to the full protocol, participant level-data, and statistical code {31c}

As the primary investigator, Dr. Tomfohr-Madsen will remain the custodian of the data collected during the trial. Data will not be released to any third party (including the funder) before trial completion and will be analyzed independently by the study team. Recognizing the importance of sharing results, data will be shared in accordance with the International Committee of Medical Journal Editors’ guidelines, which state that authors share with others the deidentified individual patient data underlying results presented in the trial reports (including tables, figures, and appendices or supplementary material) no later than 6 months after publication. Data will be made available upon request to the primary investigator.

## Oversight and monitoring

### Composition of the coordinating center and trial steering committee {5d}

Dr. Tomfohr-Madsen, as the Primary Investigator, will be responsible for the overall management of the project. She received extensive training in cognitive behavioral interventions and is responsible for training therapists in the delivery of CBT-I adapted for pregnant women. Dr. Madsen is the Director of the University of Calgary Psychology Clinic and is responsible for training and supervising the administration of diagnostic interviews. Dr. Giesbrecht is an expert in developmental psychobiology and will assist with the data collection, management of sleep data, and data analysis. Dr. Campbell is an experienced behavioral sleep medicine specialist and will provide clinical support regarding the establishment of therapy protocols and ongoing consultation regarding fidelity to the CBT-I treatment protocol. Dr. Metcalfe is an expert in maternal health and will assist with study recruitment, access to delivery data from the Alberta Perinatal Health Program, and knowledge dissemination. Dr. Keys is a registered nurse with expertise in infant sleep medicine who has contributed to the treatment protocol and will assist in tailoring the CBT-I intervention for postpartum participants. Dr. Williamson is a biostatistician and will assist with final data analyses. Kelly Mrklas is a health care system knowledge translation and implementation scientist and will assist with dissemination, and potential policy/programming change related to trial findings.

### Composition of the data monitoring committee, its role, and reporting structure {21a}

The trial will be monitored by the study investigators. The primary investigator will meet bi-weekly with the research coordinator to review recruitment, randomization, and assessment completion. A formal data monitoring committee is not needed for the current trial given its relatively small sample size (62 participants), short duration (5 weeks of psychological intervention), and known minimal risks (no anticipated discomforts or inconvenience to participants, some may experience mild fatigue).

### Adverse event reporting and harms {22}

No adverse events were reported in our pilot studies, and to our knowledge, no known risks to pregnant women are associated with CBT-I. To ensure participant safety, we have a treatment algorithm in place to triage participants reporting adverse events to appropriate acute or long-term treatment. The primary investigator will check in weekly with the research coordinator and therapists to ensure that the study participants’ concerns and questions are responded to appropriately.

The University of Calgary’s standard procedures will be followed for reporting adverse events and protocol violations/deviations to the Conjoint Health Research Ethics Board and privacy breaches to the Access and Privacy Coordinator.

### Frequency and plans for auditing trial conduct {23}

There are no plans for independent auditing of trial conduct. The Conjoint Health Research Ethics Board may request an audit.

### Plans for communicating important protocol amendments to relevant parties (e.g., trial participants, ethical committees) {25}

Amendments to the protocol will be submitted and reviewed for approval by the Conjoint Health Research Ethics Board at the University of Calgary. If the amendment requires the revised information to be provided to participants currently enrolled in the study trial, this is communicated through a consent addendum. For example, COVID-19 was declared a global pandemic in March 2020, part way through the trial; therefore, the protocol was modified to provide the intervention via telehealth through a secure virtual platform, following local regulations; and participants received a telehealth consent form addendum.

### Dissemination plans {31a}

Using a multifaceted approach, research findings will be disseminated to academic and nonacademic audiences. To communicate research findings to the public and knowledge users, we will share key findings through our study and lab website and through social media channels. Second, we will work with Media Relations at the Alberta Children’s Research Institute (ACHRI) and the University of Calgary to disseminate findings through local and national media outlets. We will use existing community contacts to host presentations that share findings with specific perinatal and parenting groups and educators. We will disseminate our research with academic and professional audiences via presentations at conferences and publication of findings in high impact journals, consistent with the Tri-Agency Open Access Policy. In addition, if trial findings substantiate the efficacy of CBT-I in pregnancy, we will coordinate with our applied health research partners (e.g., Strategic Clinical Networks) to explore opportunities for health system integration through implementation and scale-up funding.

## Discussion

CBT-I delivered in pregnancy has the potential to reduce symptoms of insomnia and depression and could lead to reduced risk of preterm birth, all of which can minimize risk of negative maternal and child health and developmental consequences in the short (e.g., infant death) and long terms (e.g., developmental delays). This RCT builds on the successful open pilot trial conducted by our team and will provide further evaluation of a novel evidence-based treatment for pregnancy-related insomnia, which can be widely disseminated and used to treat individuals that are most in need of intervention. Moreover, the challenges presented by the COVID-19 pandemic and ensuing physical distancing measures led to modifications in the protocol to ensure treatment could continue to be provided, via telehealth. This will be the first study to investigate the delivery of CBT-I via telehealth (videoconferencing) during pregnancy. Findings will enhance understanding of pregnancy-related sleep problems, as well as means by which to improve the health and sleep of both mothers and their children.

## Trial status

The trial protocol was last updated on April 15, 2020 (Version 4). Recruitment began on July 24, 2019, and was anticipated to finish in January 2021 (with estimated study completion in January 2022 for collection of all primary outcomes). However, there was a significant influx of interest in participating in the trial after COVID-19 was declared a global pandemic in March 2020, when the protocol was modified to offer the intervention via telehealth due to social distancing guidelines, leading to recruitment finishing earlier than anticipated on October 21, 2020. Given the ensuing increased study operation demands, we were not able to submit the protocol sooner. The last patient visit is now estimated to be completed in November 2021.

## Appendix

### Structure of cognitive-behavioral therapy for pregnant women with insomnia

Session1: Introduction

1. Discuss the overview of the CBT-I program and the basic principles of how to cope with sleep problems.
2. Help participants set realistic goals for what they can get out of the program.
3. Discuss the importance of self-monitoring.
4. Encourage participants to practice using the Daily Sleep Diary.

Session 2: Sleep basics and sleep consolidation

1. Psychoeducation about the basic biology of sleep and to become familiar with terms such as sleep efficiency, sleep stages, and slow-wave sleep.
2. Recognize and understand the controllable and uncontrollable factors that affect sleep, such as alterations of hormonal changes that can affect sleep quality and quantity during pregnancy
3. Present the rationale behind the behavioral strategies for managing sleep disturbances.
4. Discuss about the importance of mastering the technique of sleep consolidation.

Session 3: Stimulus control

1. Review progress using sleep consolidation procedures and help adjust the sleep window.
2. Learn stimulus control procedures as a means of re-establishing a positive association between bed and sleeping.
3. Talk about steps to take control of sleep problems.

Session 4: Cognitive therapy

1. Discuss self-defeating thoughts, negative feelings, and poor sleep behaviors.
2. Review biased ways of thinking about sleep, signs that self-talk may be negative and self-defeating and the categories of negative self-talk.
3. Examine attitudes and beliefs about sleep.
4. Discuss the general techniques for managing stress.
5. Identify and challenge participants’ negative self-talk about their sleep.

Session 5: Problem solving and relapse prevention

1. Talk about problem solving to cope with sleep disturbances (S.O.L.V.E. Technique).
2. Emphasize the importance of maintaining treatment gains and continuing to work towards better sleep.
3. Discuss about effective ways to cope with relapse, especially in the postpartum period.
4. Discuss infant sleep and how to best establish health habits for infants in the postpartum period.

## Abbreviations

BISQ: Brief Infant Sleep Questionnaire
CBT: Cognitive behavioral therapy
COVID-19: Coronavirus disease 2019
DIAMOND: Diagnostic Interview for Anxiety, Mood, and OCD and Related Neuropsychiatric Disorders
DSM: Diagnostic and Statistical Manual of Mental Disorders
EPDS: Edinburgh Postnatal Depression Scale
GEE: Generalized estimated equations
ISI: Insomnia Severity Index
ITT: Intent-to-treat
ORBIT: Obesity Related Behavioral Intervention Trials
PPD: Postpartum depression
PSA: Pregnancy Specific Anxiety
PSQI: Pittsburgh Sleep Quality Index
RCT: Randomized controlled trial
RLS: Restless legs syndrome
SCISD: Structured Clinical Interview for DSM-5 Sleep Disorders
SDB: Sleep-disordered breathing
STAI: Strait Trait Anxiety Inventory
TAU: Treatment as usual
